# Changes in total and per-capital ecosystem service value in response to land-use land-cover dynamics in north-central Ethiopia

**DOI:** 10.1038/s41598-024-57151-6

**Published:** 2024-03-19

**Authors:** Emiru Birhane, Emnet Negash, Tesfaye Getachew, Hailemariam Gebrewahed, Eskinder Gidey, Mewcha Amha Gebremedhin, Paidamwoyo Mhangara

**Affiliations:** 1 Department of Land Resources Management and Environmental Protection, Institute of Climate and Society, Mekelle University, Mekelle, Ethiopia; 2 Faculty of Environmental Sciences and Natural Resource Management, Norwegian University of Life Sciences (NMBU), Ås, Norway; 3 Department of Geography, Ghent University, Ghent, Belgium; 4 Institute of Geo-Information and Earth Observation Sciences, Mekelle University, Mekelle, Ethiopia; 5 School of Geography, Archaeology and Environmental Studies, Faculty of Science, University of the Witwatersrand, Johannesburg, South Africa; 6 Department of Land Resources Management and Environmental Protection (LaRMEP), College of Dryland Agriculture and Natural Resources, Mekelle University, P.O Box: 231, Mekelle, Tigray Ethiopia; 7 School of Geography, Archaeology and Environmental Studies, Faculty of Science, University of Witwatersrand, Johannesburg, Private Bag: 2050 Gauteng South Africa; 8 Department of Water Resources, University of Twente, Enschede, The Netherlands

**Keywords:** Benefit transfer, Ecosystem services, Landcover change, Mountain region, Ecosystem services, Ecosystem ecology

## Abstract

Ecosystems provide a wide range of services crucial for human well-being and decision-making processes at various levels. This study analyzed the major land cover types of north-central Ethiopia and their impact on total and per-capita ecosystem service value (ESV). The ESV was estimated using the benefit-transfer method along the established global and local coefficient values for the periods 1973, 1986, 2001, 2016, and 2024. The findings show that agricultural lands continued to expand at a rate of 563.4 ha year^−1^, at the expense of forests and grasslands. As a result, the total ESV of the study area declined from $101.4 to $61.03 million and $60.08–$43.69 million, respectively. The ESV per capita was also diminished by $152.4 (37.7%) and $257 (40.6%), respectively. However, land-cover improvement during the period 2001–2016 enhanced the total and per capita ESV in the study area. Therefore, potential future research may be required to develop a valid approach for assessing the robustness and sensitivity of value coefficients for the valuation of the ESV at the landscape level.

## Introduction

Ecosystems not only enhance productivity but also, by their services, provide for human well-being, health, livelihoods, and survival^1–7^. Ecosystem services have become important in research and policymaking^8–10^, so natural capital quantification and conceptualization have received much attention. The value of global ecosystem services in 1997 was estimated to be about USD 33 trillion per year^7,10^, which is a figure higher than the global gross domestic product at the time. Ecosystem service value (ESV) estimates have varied over time with changes in determinant factors. The quality and quantity of the ESV are based on the characteristics of the surrounding ecosystems^7,11–14^. Population growth, economic development, and urban expansion are among the major causes of ecosystem service damage these days. Additionally, land cover changes are the major causes of global environmental change and sustainable development^15–18^. Anthropogenic activities have a significant impact on changes in ecosystem service values^19,20^. These changes affect all the structure, processes, and biodiversity, which in turn determine the ESV in a landscape^7,14–17^. On the other hand, change in ESV depends on the magnitude and direction of changes in land use and land cover.

There is a variation in defining what an ecosystem service represents^21^. Ecosystem services represent the direct and indirect goods and services and functions people derive and use from the ecosystem functions^2,7,22^. Ecosystem service value means the conditions and the process through which the natural ecosystem sustains and fulfils the needs of human life^22–24^. The economic values of ecosystems vary in time and space, ranging from the short-term site level to the long-term global level^25^. Previous studies quantified ESVs and their change by compiling a list of ecosystem service coefficients of biomes^7,11^ and extracted the equivalent weight factor of ecosystem service per hectare of terrestrial ecosystems and modified ESV coefficients^26^. Studies have also modified the corresponding value coefficients of ESV towards a more conservative coefficient^14,27^.

Although there are several methods to value ecosystem services, the benefits transfer method is widely used^1,14,28–30^, mainly because it is cost-effective^5,31,32^. In Ethiopia, the densely populated highlands and midlands are experiencing rapid population growth and worrying trends in land cover with increasing competition for resources^5,14,33–36^, which, in turn, degrades the ecosystem service of the landscape. In addition, there are not sufficient studies estimating the monetary value of environmental degradation in tropical drylands, including Ethiopia^37^. Hence, this study aimed to investigate the impact of land-cover changes on total and per capita ESV along the Borena landscape in north-central Ethiopia. The work aims to analyze changes in the total and per-capita ecosystem service value of a landscape mosaic in north-central Ethiopia in response to changes in land use and land-cover change. Findings would help raise public awareness of the cost of transforming natural landscapes into other land uses, which could shape ecosystem service value, provide support for sustainable policymaking, and therefore reach sustainable environmental management.

## Methods

### Study area

**Figure 1:**
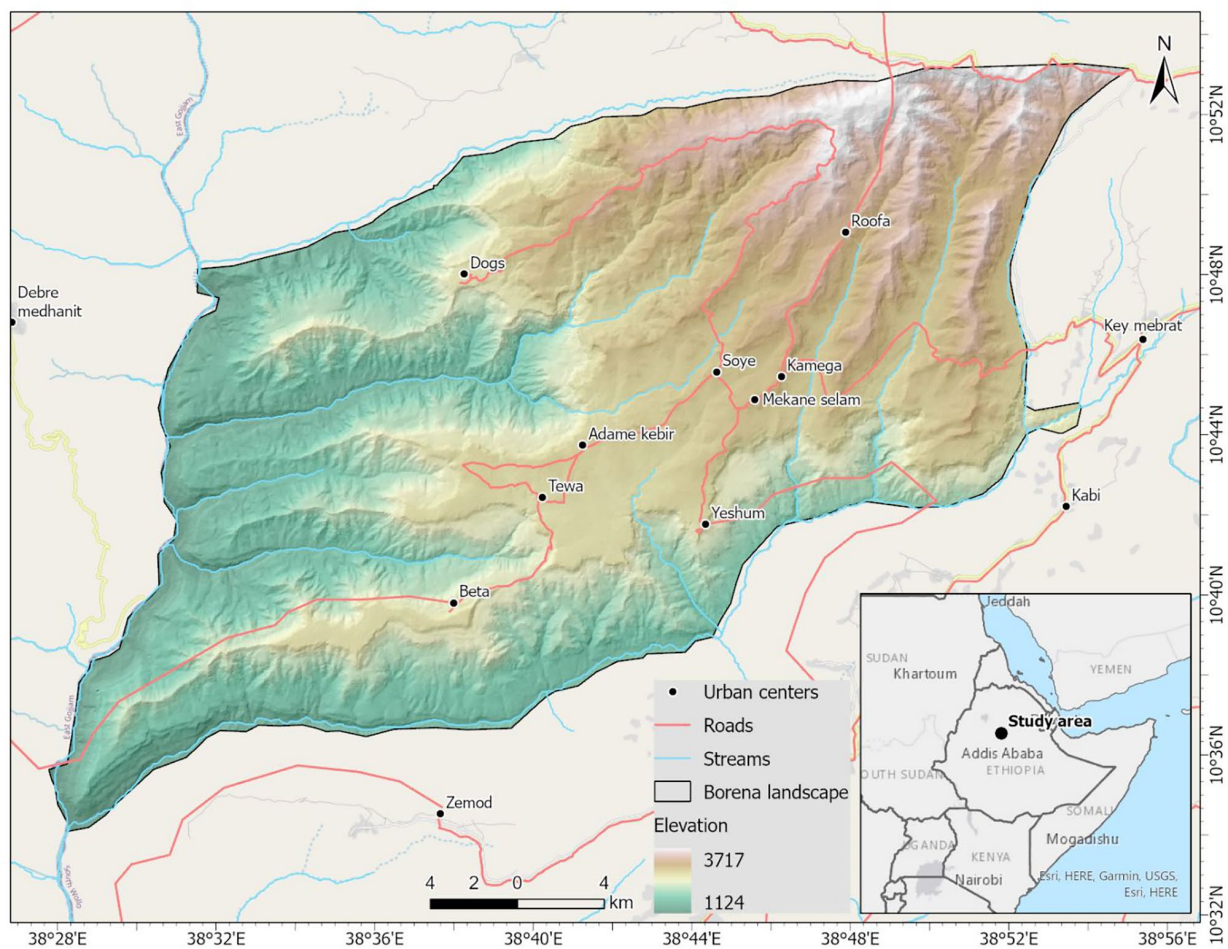
Orography and location map of the study area.

The mountainous landscape of Borena is found in the north-central Ethiopian highlands. The landscape is geographically located between 10° 30′ 0ʺ to 10° 55′ 0ʺ N and 38° 30′ 0ʺ to 38° 55′ 0ʺ E (Fig. 1). The district, with a total area of 93,856 hectares, is found about 180 km southwest of Dessie town in the South Wollo administrative zone of Amhara National Regional State, Ethiopia. The study area is a mountainous region characterized by diverse topographic conditions with an elevation between 1124 and 3717 m above sea level. Mountains and highly dissected terrains with steep slopes characterize the upstream part of the landscape on the northeast side, while up-and-down topography and gentle slopes characterize the landscape downstream toward the west and southwest side^32^.

The complex topography of the region as well as the seasonal migration of the Intertropical Convergence Zone (ITCZ) controls the climate of Ethiopia in general^38^ and the study area. The study area has received a total annual rainfall value between 889 and 1500 mm every year. The highest rainfall was received between June and September, but short rains occurred during March, April, and May. The mean annual temperature varies between 14 and 19 °C^32^. The upper northwestern part of the study area is known for its minimum temperature that results in the prevalence of a cold, locally Wurch type of climate, while the southwestern part of the district has the highest temperature characterized by hot, locally *Kolla* climate conditions.

### Data collection, processing, and analysis

#### Land use land-cover dynamics

Land-cover datasets were required to evaluate changes in land cover as well as the ESV of the landscape. Accordingly, these land use and land-cover information for the years 1973, 1986, 2001, 2016, and 2024 (Table 1) were extracted from Landsat satellite images downloaded from the USGS website (https://earthexplorer.usgs.gov), using an object-based classification Kindu et al.^35^ in eCognition and machine learning models such as Random Tree in ESRI ArcGIS Pro 3.2.0, mainly that because regular quantification, monitoring, modeling, analysis, and mapping of the spatial and temporal dynamics of land-use and land-cover change (LULCC) is required to acquire knowledge of the real-time processes, diversity, and change that occurs on the land surface^39^. During the classification procedure, spectral differences between the various types of land use were taken into consideration^39^. After that, the change detection analysis was performed using overlay analysis^40^. Satellite images for a dry month were considered for analysis to avoid seasonal effects such as phenological effects. Land-cover information was then extracted using an object-based classification in eCognition and ESRI ArcGIS Pro 3.2.0.

**Table 1:** Satellite images designation.

Images	Path	Row	Sensor ID	Pixel size in meters	# Bands	Acquisition date
Landsat 1	181	052	MSS	60 × 60	4	1/31/1973
	181	053				
Landsat 4–5	169	052	TM	30 × 30	7	1/28/1986
	169	053				
Landsat 7	169	052	ETM +	30 × 30	9	1/13/2001
	169	053				
Landsat 8	169	052	OLI/TIRS	30 × 30	11	2/16/2016
	169	053				
Landsat 9	169	052	OLI/TIRS	15 × 15 (after enhancement)	9	2/11/2024
	169	053	OLI/TIRS		9	2/11/2024

The reference years for land cover and ESV were purposely selected to detect major socio-political and environmental events in the region. In Ethiopia, 1973–74 was a turning point from an imperial to a socialist-oriented military government. In 1985/86, there was a serious drought in Ethiopia, especially “the Wollo and Tigray famine,” including the study area. Government-led large-scale environmental rehabilitation activities in Ethiopia were introduced in the mid-1970s, with several success stories and failures observed^41^. Since 2000/01, the Federal Democratic Republic of Ethiopia (FDRE), in its successive 5 year national development plans, has further emphasized natural resource management. The year 2024 was included to mark the recent image of the study area.

#### Ecosystem service value estimation

There are several direct and indirect ecosystem service valuation approaches^30,41,42^. Direct service valuation methods are essentially the exchange values that ecosystem services have in trade, mainly applicable to the ‘goods’ but also some information functions and regulation functions. Such methods are highly accurate and precise in their valuation of ecosystem services, but they are not cost-effective. On the other hand, there is a need to resort to indirect means of service value assessment when there are no explicit markets for services. These methods consider the willingness to pay and accept the availability or loss of these services^41^.

In this study, the benefit transfer method^30^, an indirect method, was used to extrapolate the ESV to the landscape. The method uses an economic estimate of the value of market and non-market services adopted for the analysis of an existing single study or group of studies, carried out to estimate the ESV of a similar location in the absence of site-specific valuation data^7,30,32,43^. The ESV estimation was performed considering a global coefficient adopted from Costanza et al.^7^ and coefficients locally adjusted for the Ethiopian highlands adopted from Kindu et al.^14^. The benefit transfer method was selected for its cost-effective advantage^5,31^. As this method is a technique to estimate the economic value of the environment based on the value of another completed study, the similarities between the study site and the policy site, i.e. an area where coefficient values are adopted, as well as the quality of the original study, are crucial. In this study, both the study site and policy site found in the Ethiopian highlands showed similar characteristics (Table 2). It should be noted that ecosystem service value estimates based on indirect methods such as benefit transfer are indicative and not as precise as direct methods. These highlights suggest that direct methods should be employed for a more precise valuation of ecosystem services. Moreover, transferring the economic value of an environment based on the value of another study mostly suits the service functions that were accounted for in earlier similar studies. Service functions that are new to the study area or functions that have not been included in earlier studies^7,14^, if any, remain unaccounted for.

**Table 2:** Characteristics of study site and policy site.

Characteristics	Borena landscape (study site)	Munessa–Shashemene landscape (Kindu et al.^14^)
Absolute location	7° 20′ to 7° 35′ N and 38° 39′ to 38° 59′ E	10°45′ to 10°53′ N and 38°28′ to 38°54′ E
Mean annual Temp	15 °C	14–15 °C
Rainfall/year	1200 mm	889–1500 mm
Elevations	1500–3400 m	1008–3696 m
Area coverage	1091 km^2^	938 km^2^

The quantification of the ESV and their change has been based on the proposed list of service value coefficients^7^, for biomes and estimated global ESVs^14,30,43^. The same method was applied in this study, using these global coefficients^7^, and a local conservative value coefficient^14^. A vigorous study by Costanza et al.^7^ is among the earliest studies to estimate global ESV and develop global coefficient values, while Kindu et al.^14^ estimated ESV of a natural forest ecosystem in Ethiopia and developed local coefficients. Tables 3 and 4 below show global and local coefficient values, respectively, for 17 individual service functions on four major service categories. The mean economic value of ecosystem service functions per unit area was estimated using existing mathematical equations adopted from Costanza et al.^7^ and Xie et al.^29^.

**Table 3:** Global coefficients ($USD ha^−1^ year^−1^), adopted from Costanza et al.^7^.

Ecosystem service	Cultivated-land	Grassland	Natural forest	Plantation forest	Waterbodies
Cultural service		2	114	114	230
Cultural			2	2	
Recreation		2	112	112	230
Provisioning service	54	67	396	396	2158
Food production	54	67	32	32	41
Genetic resources			41	41	
Raw material			315	315	
Water supply			8	8	2117
Regulating services	24	149	566	566	6110
Biological control	24	23			
Climate regulation			223	223	
Disturbance regulation			5	5	
Erosion control		29	245	245	
Gas regulation		7			
Water regulation		3	6	6	5445
Water treatment		87	87	87	665
Supporting service	14	26	932	932	
Habitat/refugia					
Nutrient cycling			922	922	
Pollination	14	25			
Soil formation		1	10	10

**Table 4:** Local conservative coefficients ($USD ha^−1^ year^−1^), adopted from Kindu et al.^14^.

Ecosystem service	Cultivated land	Grassland	Natural forest	Plantation forest	Waterbodies
Cultural service		0.8	6.8	6.8	69
Cultural			2	2	
Recreation		0.8	4.8	4.8	69
Provisioning service	187.56	117.45	132.24	132.24	2158
Food production	187.56	117.45	32	32	41
Genetic resources			41	41	
Raw material			51.24	51.24	
Water supply			8	8	2117
Regulating services	24	149	628.68	628.68	5876.5
Biological control	24	23			
Climate regulation			223	223	
Disturbance regulation			5	5	
Erosion control		29	245	245	
Gas regulation		7	13.68	13.68	
Water regulation		3	6	6	5445
Water treatment		87	136	136	431.5
Supporting service	14	26	218.97	218.97	
Habitat/refugia			17.3	17.3	
Nutrient cycling			184.4	184.4	
Pollination	14	25	7.27	7.27	
Soil formation		1	10	10	

The mean economic value of ecosystem services per unit area was estimated using the following equations established by Costanza et al.^7^ and Xie et al.^29^:

1 $${\text{ESV}}=\sum_{{\text{k}}}\sum_{{\text{f}}}{{\text{A}}}_{{\text{k}}}\times {{\text{VC}}}_{{\text{kf}}},$$

2 $${{\text{ESV}}}_{{\text{f}}}=\sum_{{\text{k}}}{{\text{A}}}_{{\text{k}}} \times {{\text{VC}}}_{{\text{kf}}},$$

3 $${{\text{ESV}}}_{{\text{k}}}=\sum_{{\text{f}}}{{\text{A}}}_{{\text{k}}}\times {{\text{VC}}}_{{\text{kf}}},$$

where ESV = total ecosystem service value of the landscape, ESV_f_ = value of ecosystem service function type “f”, ESV_k_ = ecosystem service value of land cover category “k” and ecosystem service function type “f,” AK = Area (hectare) of land use category “k”, VC_kf_ = value coefficient of “f” ($US ha^−1^ year^−1^) for each land cover using unit area ecosystem service value^7^.

#### Changes in ecosystem service value per capita

The ESV per capita calculation is important to show the relationship between the ESV and population size and growth. A similar study by Zhou et al.^44^ used the same method to indicate the relationship between ESV and size of the population. The ESV per capita was calculated using the following equation:

4 $${\text{Ave}}\left({\text{ESV}}\right)=\frac{{\text{ESV}}}{{\text{N}}}=\sum_{{\text{i}}=1}^{{\text{n}}}\frac{{{\text{VC}}}_{{\text{kf}}}}{{\text{N}}} \times {{\text{A}}}_{{\text{k}}}$$

where Ave (ESV) is the amount of ecological service per capita, N is population, and the definition of the other parameters in the formula remains the same as in Eqs. (1), (2), (3) above. Additionally, the 2024 population growth of the study area was estimated as follows (Eq. 5):

5 $$x\left(t\right)=x0\times \left(1 + r\right)t,$$

where $x0$ = Initial Population, (r) = population growth rate (i.e. 2.3%), t = number of years (t).

#### Ecosystem sensitivity

The sensitivity coefficient of economics has been recommended for ranking the importance of land–cover classes based on their contribution to the total ESV^45^. Below is the mathematical algorithm:

6 $${\text{CS}}=\frac{({\text{ESV}}-{{\text{ESV}}}_{{\text{i}}})/{{\text{ESV}}}_{{\text{i}}}}{({{\text{VC}}}_{{\text{jk}}}-{{\text{VC}}}_{{\text{ik}}})/{{\text{VC}}}_{{\text{ik}}}} ,$$

where CS is the coefficient of sensitivity, ESV is the total ecosystem service value, VC is the value coefficient; and i and j represent the initial and adjusted values of the land use type, respectively.

The value coefficient (VC) of each land-cover class is adjusted by + 50% in case large enough shifts up to that magnitude occur that could affect the global average values for ecosystem services that de Groot et al.^41^ provide and the change of ESV measured. Until recently, the elasticity coefficient has been widely used in assessing the robustness and sensitivity of ecosystem service values^14,44,45^. The method assumes that if CS > 1, then the estimated ESV is elastic, i.e. highly sensitive to changes in VC_jk_. Whereas, if CS < 1, the estimated ESV is inelastic, i.e. not sensitive to changes in VC_jk_. A vigorous study by Aschonitis et al.^46^ proved that CS values of the common approach are always in the range between 0 and 1. This shows that the approach is being erroneously applied and interpreted. Therefore, in this study, the method is only considered for ranking the importance of various land-cover classes based on their contribution to the total ESV, as per the recommendation of recent studies^32,46^.

## Results and discussion

Seven major land-cover classes were identified in the landscape of about 93,856 hectares. Generated land-cover maps were acceptable^47^, with an overall accuracy of 88.6%, and the producer’s and user’s accuracy for each land-cover class showed at least 75% and 80%, respectively. Besides, cultivated lands comprised the highest area coverage of the landscape, followed by plantation forest, grassland, natural forest, bare land, water bodies, and settlements, respectively (Table 5). In 1973, plantation forests had the highest share (45%) until it was replaced by cultivated land in 1986 (50.75%), a situation that continued until 2024 (61%). This indicates that agricultural expansion at the expense of forest cover is common in the landscape^19^.

**Table 5:** Land-cover classes area (ha) and their proportion (%) over time from 1973 to 2024.

Year	Bare land		Cultivated land		Grassland		Natural forest		Plantation forest		Settlement		Water body	
	(ha)	(%)	(ha)	(%)	(ha)	%	(ha)	(%)	(ha)	(%)	(ha)	(%)	(ha)	(%)
1973	423.2	0.45	35,596.5	37.9	11,614.9	12.4	3415.5	3.64	42,250.4	45.00	113.4	0.12	442.2	0.47
1986	760.6	0.81	47,631.7	50.75	11,254	12.0	3038.6	3.24	30,645.2	32.65	124.7	0.13	401.1	0.43
2001	853.1	0.91	59,188.5	63.06	8151.9	8.69	2291.5	2.44	22,785.6	24.28	189.7	0.20	395.8	0.42
2016	607.9	0.65	59,051	62.92	4443.6	4.73	3088.9	3.29	25,635.2	27.31	578.9	0.62	450.4	0.48
2024	5387.3	5.74	57,311.8	61.06	3091.8	3.29	4408.2	4.70	21,732.89	23.16	1005.1	1.07	919.8	0.98
Av	661.2	0.71	50,366.9	53.66	8866.1	9.45	2958.6	3.15	30,329.1	32.31	251.68	0.27	422.4	0.45
CV	4.21		563.45		− 172.6		− 11.60		− 394		10.29		0.18	
R^2^	0.17		0.87		0.93		0.21		0.73		0.75		0.01	
p*	0.58		0.07		0.04		0.55		0.15		0.13		0.88	

*CV* coefficient of variation in hectare per year.

*Significance level at *p* = 0.05.

**Figure 2:**
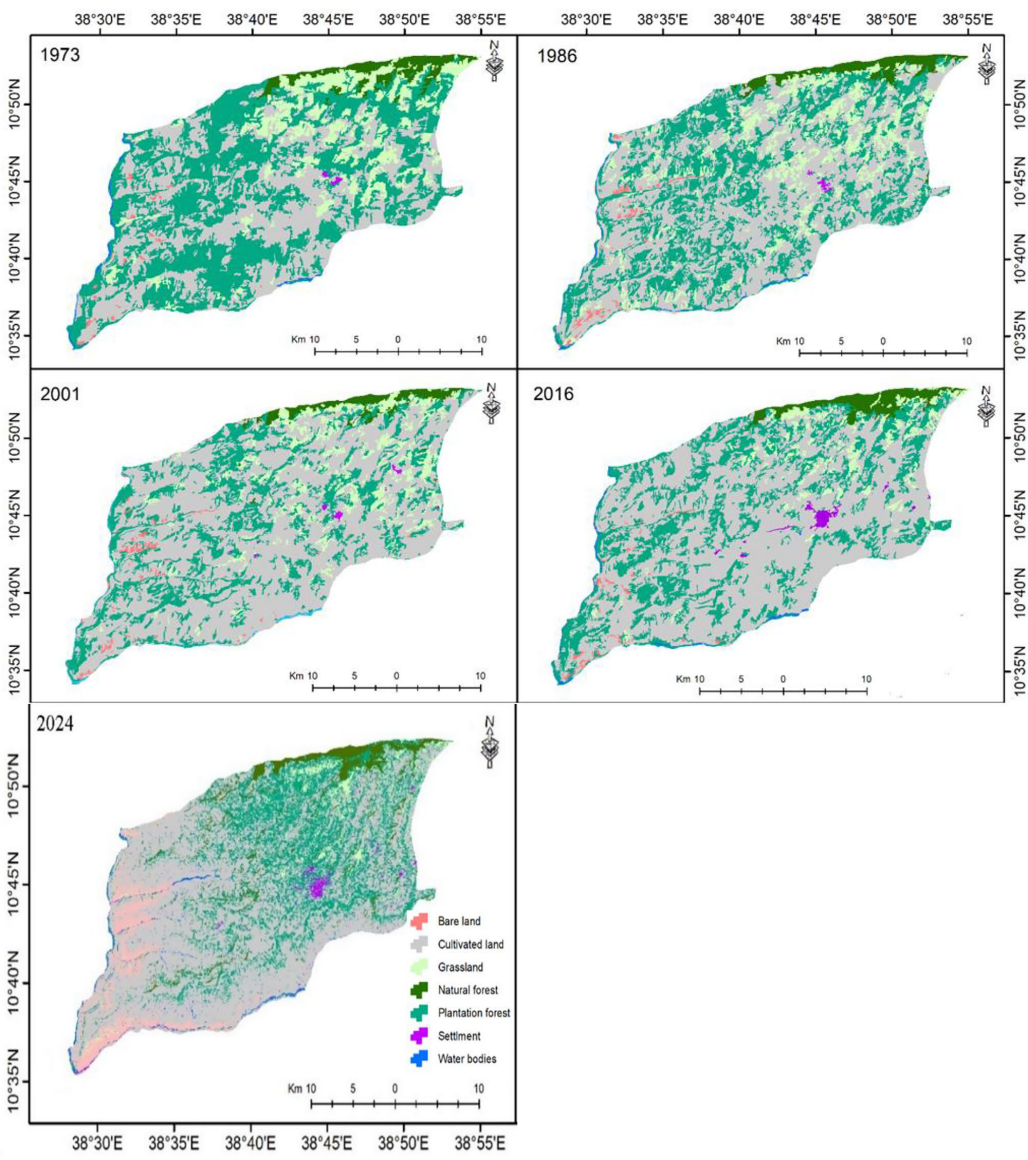
The land covers dynamics during the period between 1973 and 2024.

Moreover, although all land–cover classes were dynamic, significant parts of the landscape’s natural and plantation forests were increasingly deforested during the study period. Cultivated land kept expanding at a coefficient of 563.4 ha year^−1^, followed by settlement (10.29 ha year^−1^), bare land (4.21 ha year^−1^), and water body (0.18 ha year^−1^). On the other hand, significant parts of the landscape’s plantation forest have been threatened at a coefficient of 394 ha year^−1^, followed by grassland (172.6 ha year^−1^), and natural forest (11.6 ha year^−1^), respectively (Table 5). Despite the long–term deforestation and forest degradation from 1973 to 2001, forest cover in the landscape has improved from 26.72% in 2001 to as high as 27.8% in 2024. Settlement is the highest increment percentage, with about 410.5% raised (Table 5 and Fig. 2).

In agreement with this study, synonymous studies also noted that there had been an evident agricultural expansion in northern Ethiopia^39,48–50^. Increments in agricultural lands and settlement areas, however, severely threatened significant areas of forest cover, including grasslands in the landscape. Deforestation and forest degradation endanger the forest cover and the ecosystem's biodiversity. Although there are spatial and temporal inefficiencies, woodlands have expanded in recent years following afforestation and reforestation efforts^39,51–53^.

### Changes in ecosystem service value

**Figure 3:**
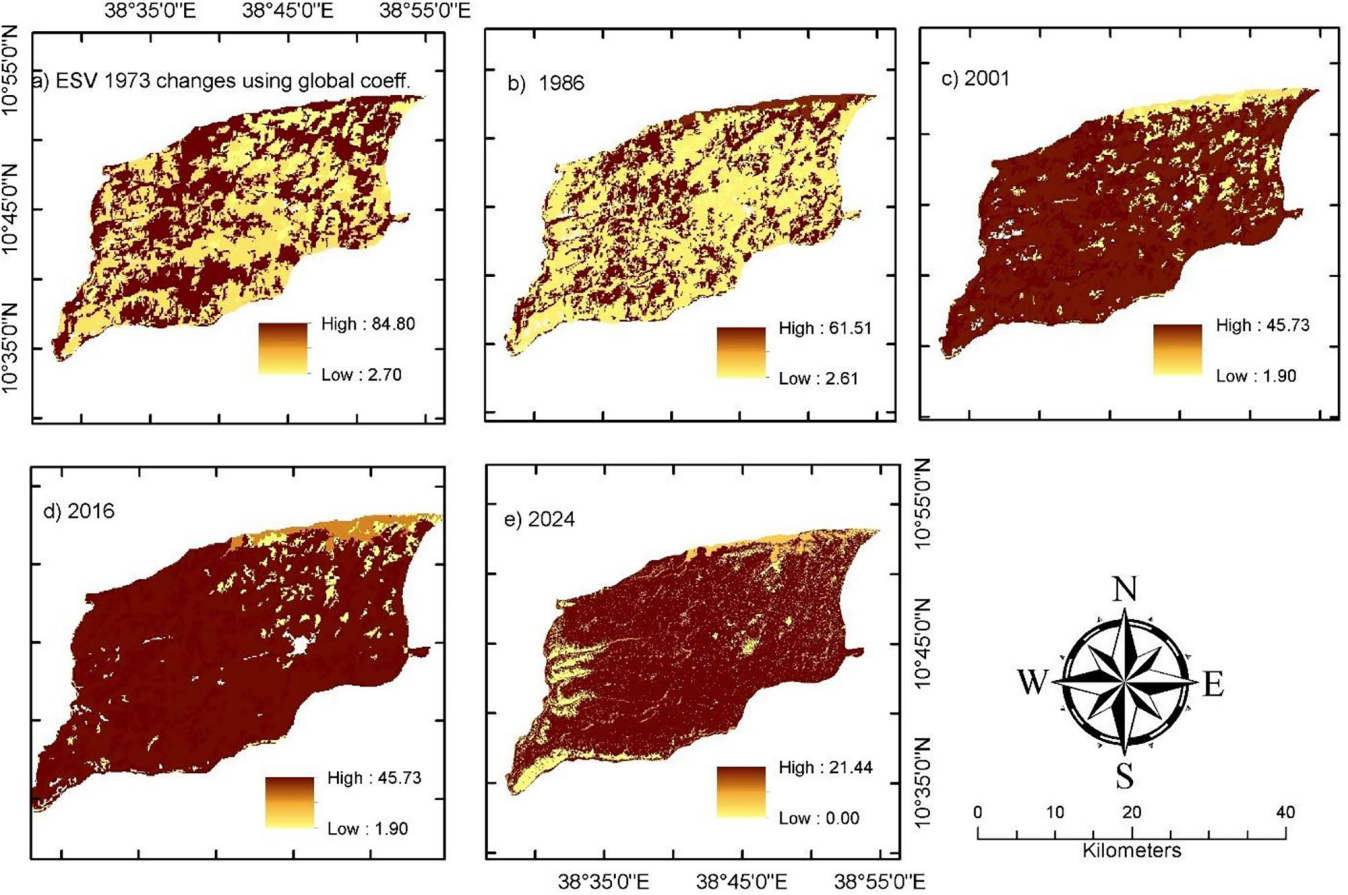
Ecosystem service value ($USD ha^−1^ year^−1^) using local (upper) and global (lower) coefficients.

The total ESV in the landscape varied between $47.08 and $101.4 million and $43.69 and $66.27 million using global and local conservative coefficients, respectively (Tables 6, 7). The highest total ESV estimate over the landscape was $101.4 million observed in 1973, followed by $78.01 million (1986) and $67.94 million (2016), and the least value was $47.08 million observed in 2001 using global coefficients. Similarly, the highest ESV estimate based on the local conservative coefficients was $66.27 million observed in 2024, followed by $60.08 million (1973) and $46.61 million (2016), and the least value was $43.69 million observed in 2001 (Table 6). This clearly showed a significant decline in ESV estimates over both global and local coefficients (Fig. 3). Among all land-cover classes in the Borena landscape, plantation forests showed the highest ESV, accounting for between $43.62 and $84.8 million, respectively, using the local and global coefficients. Bare land and settlement appeared to have the least ESV consistently throughout both global and local coefficients during the study period (Table 6). This is a function of the coefficient value equivalent to these land-cover classes.

**Table 6:** ESV estimates (million $USD year^−1^) using local (a) and global (b) coefficients.

Coefficient	Land-cover	1973		1986		2001		2016		2024	
		Mil. $	%	Mil. $	%	Mil. $	%	Mil. $	%	Mil. $	%
a	Bare land	0.00	0.00	0.00	0.00	0.00	0.00	0.00	0.00	0.00	0.00
	Cultivated land	8.03	13.36	10.74	21.26	13.35	30.56	13.32	28.57	5.27	7.96
	Grassland	3.41	5.67	3.30	6.53	2.39	5.47	1.30	2.80	0.72	1.08
	Natural forest	3.37	5.61	3.00	5.93	2.26	5.17	3.05	6.54	8.85	13.35
	Plantation forest	41.69	69.39	30.24	59.84	22.48	51.46	25.29	54.26	43.62	65.82
	Settlement	0.00	0.00	0.00	0.00	0.00	0.00	0.00	0.00	0.00	0.00
	Water bodies	3.58	5.96	3.25	6.43	3.21	7.34	3.65	7.83	7.82	11.79
	Total	60.08	100	50.53	100	43.69	100	46.61	100	66.27	100
b	Bare land	0.00	0.00	0.00	0.00	0.00	0.00	0.00	0.00	0.00	0.00
	Cultivated land	3.27	3.23	4.38	5.62	5.45	8.92	5.43	8.00	12.93	27.46
	Grassland	2.69	2.66	2.61	3.35	1.89	3.1	1.03	1.52	0.91	1.93
	Natural forest	6.85	6.76	6.10	7.82	4.60	7.54	6.20	9.12	4.35	9.24
	Plantation forest	84.8	83.64	61.5	78.85	45.73	74.93	51.45	75.73	21.44	45.55
	Settlement	0.00	0.00	0.00	0.00	0.00	0.00	0.00	0.00	0.00	0.00
	Water bodies	3.76	3.71	3.41	4.37	3.36	5.51	3.83	5.63	7.45	15.83
	Total	101.4	100	78.01	100	61.03	100	67.94	100	47.08	100.00

**Table 7:** Estimated ecosystem functions (ESV_f_ in million $USD) by service category.

Services functions	Using global coefficients						Using local coefficients					
	1973	1986	2001	2016	2024	Change	1973	1986	2001	2016	2024	Change
Cultural service	5.33	3.96	2.97	3.39	3.20	− 2.13	0.35	0.27	0.2	0.23	0.24	− 0.11
Cultural	0.09	0.07	0.05	0.06	0.05	− 0.04	0.09	0.07	0.05	0.06	0.05	− 0.04
Recreation	5.24	3.89	2.92	3.33	3.15	− 2.09	0.26	0.2	0.15	0.17	0.19	− 0.07
Provisioning service	21.73	17.53	14.53	15.8	15.43	− 6.3	15.03	15.58	16.57	16.37	17.10	2.07
Food production	4.18	4.42	4.56	4.42	3.97	− 0.21	9.52	11.35	12.88	12.54	12.54	3.02
Genetic resources	1.87	1.38	1.03	1.18	1.07	− 0.80	1.87	1.38	1.03	1.18	1.07	− 0.80
Raw material	14.38	10.61	7.9	9.05	8.23	− 6.15	2.34	1.73	1.28	1.47	1.34	− 1.00
Water supply	1.3	1.12	1.04	1.18	2.16	0.85	1.3	1.12	1.38	1.18	2.16	0.85
Regulating service	30.98	24.34	19.25	21.1	82.50	51.52	33.9	26.35	20.4	22.77	23.68	− 10.22
Biological control	1.12	1.4	1.61	1.52	0.07	− 1.05	1.12	1.4	1.61	1.52	1.45	0.33
Climate regulation	10.18	7.51	5.59	6.41	5.83	− 4.35	10.18	7.51	5.56	6.41	5.83	− 4.35
Disturbance regulation	0.23	0.17	0.13	0.14	0.13	− 0.10	0.23	0.17	0.13	0.14	0.13	− 0.10
Erosion control	11.53	8.58	6.38	7.17	6.49	− 5.03	11.53	8.58	6.08	7.17	6.49	− 5.03
Gas regulation	0.08	0.08	0.06	0.03	0.02	− 0.06	0.71	0.54	0.4	0.42	0.38	− 0.33
Water regulation	2.72	2.42	2.33	2.64	5.17	2.46	2.72	2.42	2.33	2.64	5.17	2.46
Water treatment	5.12	4.18	3.15	3.19	64.78	63.13	7.41	5.73	4.29	4.47	4.22	− 3.19
Supporting service	43.36	32.18	24.28	27.6	25.25	− 18.11	10.8	8.33	6.52	7.24	6.61	− 4.19
Habitat/refugia	0.00	0.00	0.00	0.00	0.00	0.00	0.79	0.58	0.43	0.5	0.45	− 0.34
Nutrient cycling	42.1	30.88	23.76	26.4	24.10	− 18.00	8.42	6.21	4.62	5.3	4.82	− 3.60
Pollination	0.79	0.95	0.26	0.94	0.88	0.09	1.12	1.19	1.21	1.15	1.07	− 0.05
Soil formation	0.47	0.35	0.26	0.29	0.26	− 0.20	0.47	0.35	0.26	0.29	0.26	− 0.20
Total	101.4	78.01	61.03	67.9	126.37	53.43	60.08	50.53	43.69	46.61	47.63	− 24.89

The total ESV loss during the study period was $13.5 million and $33.5 million using the conservative local coefficients and global value coefficients, respectively (Tables 6, 7). ESV estimates using global coefficients are higher than estimates using local conservative value coefficients. It is also noted that ESV estimates using global coefficients are up to 2.4 times higher than the local conservative value coefficients^14^. ESV estimates in the Borena landscape showed a declining trend throughout the period between 1973 and 2001. Unlike the preceding three decades, total ESV estimates in 2016 rose by $6.91 million and $2.92 million using global and local coefficients, respectively (Table 6 and Fig. 3). Moreover, the declining trend in ESV over the landscape was consistent with changes in land cover^54,55^. This implies that the declining ESV estimates during the period between 1973 and 2001 and an increment in 2024 are attributed to degradation and restoration in area coverage of plantation forest, grassland, and natural forest in the landscape, respectively^5,14,17^ witnessing a success to the recently introduced environmental protection policy. Besides, estimates during 2001 and 2016 continued to be less than average on estimates using both global and local coefficients (Table 6).

#### Land-cover change and ecosystem service value

**Figure 4:**
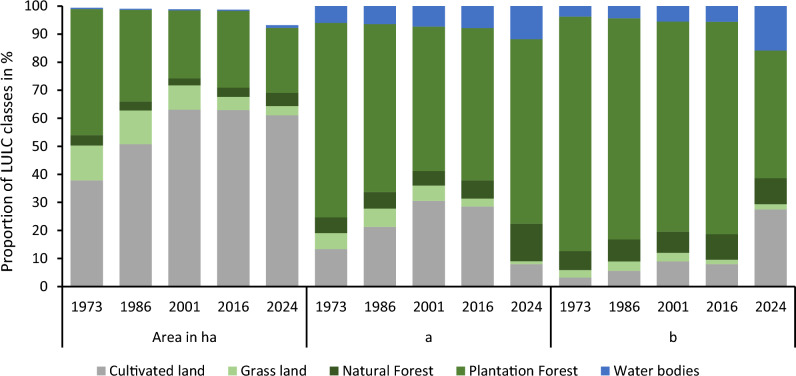
ESV contribution (%) of land-cover classes using (**a**) local and (**b**) global coefficients.

The total area coverage of plantation forests, natural forests, grassland, and water bodies consistently decreased with varying proportions over the study period between 1973 and 2001 (Table 5 and Fig. 4). An area of grassland declined threefold, and a forest area declined by almost half with a slight increment in 2024. In agreement with land cover trends, total ESV severely declined over the study period. Only using the local coefficients, ESV received from plantation forests, natural forests, and grasslands declined from about 69.39–51.46%, 5.61–5.14%, and 5.67–5.47%, respectively (Table 6). On the other hand, area coverage of plantations and natural forests showed a slight enhancement from 2001 to 2024. As a result, the total ESV of plantations and natural forests during the period 2001–2024 increased from about $50.33 million to $54.7 million and $24.74 million to $52.47 million using global and local coefficients, respectively. Although an increase has been observed, total ESV remains below average. In line with this study, similar studies showed a cumulative declining trend in ESV throughout the study period^5,14,56^. In Ethiopia, there is a loss of about USD 85 billion per year from the loss of ecosystem services due to the conversion of natural landscapes to human-impacted landscapes^37^. On the other hand, a recent study by Negash et al.^32^ showed that ecosystem service value depletion in Ethiopia is mostly associated with human habitation and therefore human-induced. Higher service value depletion in areas with high human population density is an essential indicator of the role of population pressure on land degradation and, in turn, determining ecosystem service value^57,58^. Moreover, the diminishing value of ecosystem services over time suggests they are associated with an increasing population^59^. The results would therefore mean the monetary value of human-induced environmental degradation in any landscape.

#### Estimated individual ecosystem function

The individual ecosystem function shows the contribution of each service function and category to the overall ESV during the study period. According to the estimates based on global coefficients, the supporting service category contributed to the highest share between $43.36 and $27.62 million, followed by regulating service ($30.98–$21.1 million), provisioning service ($21.73 million) and cultural service category ($5.33 million). Similarly, estimates using local coefficients in the regulating service category contributed to the highest share, accounting for $33.9–$22.77 million, followed by provisioning service ($15.03–$16.37 million), supporting service ($10.8–$7.24 million) and cultural service ($0.35–$0.23 million), respectively, during the period 1973–2016 (Table 7).

**Figure 5:**
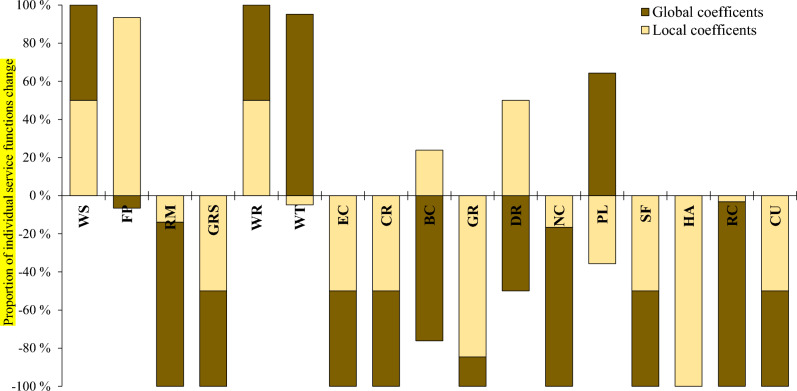
The proportion of individual service functions changes along global and local coefficient values from the period 1973 to 2024. Where *WS* water supply, *FP* food production, *RM* raw material, *GRS* genetic resource, *WR* water regulation, *WT* water treatment, *EC* erosion control, *CR* climate regulation, *BC* biological control, *GR* gas regulation, *DR* disturbance regulation, *NC* nutrient cycling, *PL* pollination, *SF* soil formation, *HA* habitat, *RC* recreation, *CU* cultural.

The provisioning service category dominated by the food production function (72.8%), based on local coefficients, exceptionally showed improvement, but all other service categories along with both global and local coefficients kept depreciating. Improvements in provisioning service categories are associated with the massive agricultural expansion throughout the landscape. Agricultural land in the Borena landscape was expanding at a coefficient of 563.45 ha year^−1^ (Table 5), and thus land availability for food production increased. This, together with the higher class’s coefficient value, contributed to the increment in the provisioning service category over the landscape, but other service categories are declining. Unlike other individual service functions, the food production function from the provisioning service category, the pollination function from the supporting service, and the biological control function from the regulating service category exceptionally showed an increasing contribution along both coefficient values (Table 7, Fig. 5). Despite a general diminishing trend in total and per-capita ecosystem service values, the results of this study exhibit an overall increment in service value received from food production and biological control functions. A prominent study of the Munessa–Shashemene landscape^14^ similarly witnessed enhanced food production, and Tolessa et al.^5^, studying ecosystem services over the Chilimo forest of West Shoa, revealed pollination function as the only function improving throughout the study period. An increase in the service value of the food production and pollination functions is attributed to the expansion of cultivated lands over the other land cover classes^59^. Keeping other factors constant, food production increases with increasing agricultural land. This explains the contrasting relations among the service functions with increasing and decreasing patterns.

### Ecosystem service value per capita

The total population in the landscape increased from about 0.13 million in 1983 to 0.17 million in 2001, 0.19 million in 2016, and 0.22 in 2024, unlike the declining trend in ESV^60^. ESV per capita estimates based on global coefficients declined from $623.45 in 1983 to $351.82 in 2001 and $298 in 2016 (Table 8). Similarly, ESV per capita estimates based on local coefficient values declined from $403.83 in 1986 to $251.86 in 2001 and $211.7 in 2024. The per capita estimates declined with the increasing population, showing an inverse relationship. Moreover, like trends in land cover and total ESV estimates (Tables 5, 6), ESV per capita also increased in 2016 relative to 2001 and decreased in 2024 (Table 8).

**Table 8:** Summary of ESV per capita in $USD using global and local coefficients.

Variable	Using global coefficients					Using local coefficients				
	1973	1983	2001	2016	2024	1973	1983	2001	2016	2024
ESV (mil. $)	101.4	78.01	61.03	67.94	126.37	60.08	50.53	43.69	46.61	47.63
Population (mil.)	*	0.13	0.17	0.19	0.22	*	0.13	0.17	0.19	0.22
ESV per capita ($)		623.45	351.82	366.45	298.00		403.83	251.86	251.4	211.7

*Population data not available.

Estimates based on local value coefficients showed that plantation forests and cultivated land in the landscape together accounted for about 82% of the total ESV per capita in 2016, whereas water bodies, natural forests, and grasslands contributed the remaining 18% only (Table 9). Similarly, estimates based on global coefficients for the same year revealed that plantation forest alone contributed about 75.7%, but natural forests, cultivated land, water bodies, and grassland altogether contributed the remaining 25%. Like total ESV, ESV per capita estimates based on global coefficient values are higher than estimates based on local conservative coefficients (Tables 6, 9)^43,61,62^.

**Table 9:** Contribution of land-cover classes to ESV per capita along (a) global and (b) local coefficients.

Coeff	Land-cover classes	ESV per capita ($USD)								
		1973	1986	%	2001	%	2016	%	2024	%
a	Bare land	–	0.00	0.00	0.00	0.00	0.00	0.00	0.00	0.00
	Cultivated land	–	35.02	5.62	31.39	8.92	29.30	8.00	23.71	7.96
	Grass land	–	20.87	3.35	10.90	3.10	5.56	1.52	3.23	1.08
	Natural forest	–	48.74	7.82	26.51	7.54	33.44	9.12	39.78	13.35
	Plantation forest	–	491.54	78.85	263.62	74.93	277.51	75.73	196.13	65.82
	Settlement	–	0.00	0.00	0.00	0.00	0.00	0.00	0.00	0.00
	Water bodies	–	27.24	4.37	19.39	5.51	20.65	5.63	35.15	11.79
	Total	–	623.42	100	351.82	100	366.46	100.00	298.0	100.0
b	Bare land	–	0.00	0.00	0.00	0.00	0.00	0.00	0.00	0.00
	Cultivated land	–	85.86	21.26	76.96	30.56	71.84	28.57	58.13	27.46
	Grassland	–	26.38	6.53	13.78	5.47	7.03	2.80	4.08	1.93
	Natural forest	–	23.96	5.93	13.03	5.17	16.44	6.54	19.56	9.24
	Plantation forest	–	241.65	59.84	129.60	51.46	136.43	54.26	96.42	45.55
	Settlement	–	0.00	0.00	0.00	0.00	0.00	0.00	0.00	0.00
	Water bodies	–	25.98	6.43	18.49	7.34	19.69	7.83	33.52	15.83
	Total	–	403.84	100.00	251.87	100.00	251.43	100.00	211.70	100.00

### Ecosystem sensitivity

The sensitivity analysis results after a + 50% adjustment in service value coefficients for all land-cover classes showed that the coefficient of sensitivity (CS) varied between 0.03 and 0.69. Plantation forests scored the highest average CS value (0.56), followed by cultivated land (0.24), water bodies (0.09), natural forests (0.06), and grassland (0.04) (Table 10). Accordingly, forest lands, i.e. plantations and natural forests alone, contributed about 65% of the total ESV on average, and all the rest contributed about 35% only. This agrees with the fact that deforestation and forest degradation have severely affected the total ESV.

**Table 10:** Changes in total ESV and coefficient of sensitivity (CS) using local value coefficients (VC + 50%).

Land-cover	1973		1986		2001		2016		2024		Average	
	Change (%)	CS	Change (%)	CS	Change (%)	CS	Change (%)	CS	Change (%)	CS	Change (%)	CS
Cultivated land	6.68	0.13	10.63	0.21	15.28	0.31	14.29	0.29	13.73	0.27	12.12	0.24
Grass land	2.83	0.06	3.27	0.07	2.74	0.05	1.40	0.03	0.96	0.02	2.24	0.04
Natural forest	2.80	0.06	2.97	0.06	2.59	0.05	3.27	0.06	4.62	0.09	3.25	0.06
Plantation forest	34.70	0.69	29.92	0.60	25.73	0.51	27.13	0.54	22.77	0.46	28.05	0.56
Water bodies	2.98	0.06	3.22	0.06	3.67	0.07	3.92	0.08	7.92	0.16	4.34	0.09
Total	50.00	1.00	50.00	1.00	50.00	1.00	50.00	1.00	50.00	1.00	50.00	1.00

Besides, the CS value for forests and grasslands declined over time, while the CS value for cultivated land and water bodies increased. The lower the CS, the lesser importance that land-cover class contributes to the total ESV, and the reverse is true. This is mainly because either the area of the land-cover class or the class coefficient value is small, thus having little effect on the estimated total ESV.

## Conclusion

The land cover of the landscape showed considerable differences in the proportion of various land cover classes during the study period, alongside alternating socio-political events. Agricultural lands and settlements grew over time, severely threatening significant forest and grassland areas, especially from 1973 to 2001. As a result, total and per capita ESV in the landscape diminished over time while the population was growing. Unlike the long-term degradation over the preceding three decades, the forest landscapes regenerated after 2001, following the introduction of the environmental protection policy in 2001. Consequently, total, and per capita ESV showed slight improvement over the past few years. Total and per capita ESV consistently declined throughout the study period with diminishing land cover, with the highest contribution received from forest lands. Thus, based on this study, land-cover dynamics in the Borena landscape have had a significant influence on the total and per capita ESV during the study period. Also, more research might be needed in the future to figure out how to directly value ecosystem services using economic methods, to estimate what might happen in the future, and to come up with a good way to check how stable and sensitive the value coefficients are. Moreover, it is important to critically analyze the drivers of land use change, their impact on ecosystem services, and the effect of policies to mitigate these impacts to restore and create resilient ecosystem services.

## Data Availability

The datasets generated during and/or analyzed during the current study are available from the corresponding author upon reasonable request.
